# Binary Pea Protein–Psyllium Hydrogel: Insights into the Influence of pH and Ionic Strength on the Physical Stability and Mechanical Characteristics

**DOI:** 10.3390/gels10060401

**Published:** 2024-06-16

**Authors:** Adonis Hilal, Anna Florowska, Ewa Domian, Małgorzata Wroniak

**Affiliations:** 1 Department of Food Technology and Assessment, Institute of Food Science, Warsaw University of Life Sciences, 02-787 Warsaw, Poland; anna_florowska@sggw.edu.pl (A.F.); malgorzata_wroniak@sggw.edu.pl (M.W.); 2 Department of Food Engineering and Process Management, Institute of Food Science, Warsaw University of Life Sciences, 02-787 Warsaw, Poland; ewa_domian@sggw.edu.pl

**Keywords:** textural properties, microrheology, physical stability, delivery system, gel induction, protein gels, food systems

## Abstract

Food hydrogels, used as delivery systems for bioactive compounds, can be formulated with various food-grade biopolymers. Their industrial utility is largely determined by their physicochemical properties. However, comprehensive data on the properties of pea protein–psyllium binary hydrogels under different pH and ionic strength conditions are limited. The aim of this research was to evaluate the impact of pH (adjusted to 7, 4.5, and 3) and ionic strength (modified by NaCl addition to 0.15 and 0.3 M) on the physical stability, color, texture, microrheological, and viscoelastic properties of these hydrogels. Color differences were most noticeable at lower pH levels. Inducing hydrogels at pH 7 (with or without NaCl) and pH 4.5 and 3 (without NaCl) resulted in complete gel structures with low stability, low elastic and storage moduli, and low complex viscosity, making them easily spreadable. Lower pH inductions (4.5 and 3) in the absence of NaCl resulted in hydrogels with shorter linear viscoelastic regions. Hydrogels induced at pH 4.5 and 3 with NaCl had high structural stability, high G’ and G” moduli, complex viscosity, and high spreadability. Among the tested induction conditions, pH 3 with 0.3 M NaCl allowed for obtaining a hydrogel with the highest elastic and storage moduli values. Adjusting pH and ionic strength during hydrogel induction allows for modifying and tailoring their properties for specific industrial applications.

## 1. Introduction

Currently, consumers are looking for products that provide health benefits beyond essential nutrition. Developing functional foods (including plant-based alternatives) has become a growing area of interest in the food industry. Developing functional hydrogels is crucial for enhancing food quality and nutritional value [1]. Hydrogels made from biopolymers like pea protein and psyllium husk have significant potential to improve the mechanical properties of food products while also serving as delivery systems for bioactive compounds [2,3,4]. The properties of these hydrogels are influenced by pH and ionic strength, and understanding their combined effects on pea protein–psyllium hydrogels is essential for creating food products with targeted textural and functional attributes while adhering to specific pH and salt content (ionic strength) requirements for various product categories [5,6,7]. This approach can allow for the fine-tuning of the gelation process, structural integrity, and bioactive compound entrapment and delivery, aligning with the growing demand for functional foods that offer enhanced texture, stability, and health benefits [8].

Such binary protein–polysaccharide food hydrogels result from combining two different biopolymers, offering advantages through precise control of polymer ratios [9]. Moreover, their properties can be customized for specific needs using, among others, induction methods [7]. They can be induced using both conventional and unconventional methods, or a combination of both. Conventional induction methods include heat treatment, pH and ion modulation, freeze–thaw cycles, and enzymatic crosslinkers [8,10]. On the other hand, unconventional induction methods include high hydrostatic pressure (HHP) [11,12] and pulsed electric field (PEF) [13]. Depending on the biopolymers and the hydrogel induction method, binary hydrogels can be formed via various mechanisms, such as ionic cross-linking, covalent cross-linking, or physical interactions [1,14]. 

During induction, pH affects the charge on biopolymer molecules, which in turn determines the degree of electrostatic interaction. Near the protein’s isoelectric point, the net charge is minimized, promoting aggregation and gel formation [15,16]. Ionic strength modulates electrostatic shielding, enhancing molecular interactions and promoting a more compact, cross-linked network [17]. Shifting pH away from the isoelectric point increases pea protein solubility, while optimal ionic strength can precipitate it, facilitating gelation [18]. Psyllium, rich in arabinoxylan, forms weak gels when used alone [19]. However, when combined with pea protein under controlled pH and ionic strength, it can enhance the gel network’s stability and water-holding capacity [20,21]. 

Pea protein derived from *Pisum sativum* L. has attracted the food industry’s interest due to its high nutritional value, good functional properties, hypoallergenic properties, and lack of concerns related to genetic modification. The proteins present in peas are salt-extractable globular storage proteins (65–80%) [22,23]. When hydrated and subjected to specific conditions (high temperature, pH above or below the isoelectric point, and increased ionic strength), these globular proteins can aggregate and form a uniform and highly crosslinked gel network [16,24]. This capability enables their application as a texturizing agent and a delivery system for bioactive ingredients. Additionally, it was proven that globular proteins, when unfolded, can bind to phenolic compounds (mainly via hydrophobic interactions and hydrogen bonds), resulting in a conjugate with higher thermal stability, antioxidant activities, and better-gelling properties [25,26,27]. On the other hand, psyllium seed husk, derived from the *Plantago ovata* plant, serves as a rich source of natural dietary fiber, primarily composed of arabinoxylan. Notably, the digestibility of arabinoxylan in humans is restricted [28,29]. Due to its hydrophilicity, psyllium mucilage has been reported to have potential use as a water-binding and moisture-retaining agent. Additionally, psyllium has been associated with beneficial effects related to satiety, cholesterol reduction, and prebiotic properties [30]. Psyllium mucilage was proven to have a weak gel-like property. When present in the small intestine, it can raise chyme viscosity, effectively slowing down the breakdown and absorption of nutrients [19]. 

Modulating the pH value and the ionic strength enables precise control over hydrogel texture and firmness, making them suitable for food products like plant-based meats [31,32,33] and dairy alternatives [34]. Additionally, pH and ionic strength are crucial for the entrapment and controlled release of bioactive compounds in functional foods, ensuring the hydrogel’s integrity and the desired release rate of bioactives [35,36]. Bioactive compounds such as vitamins, minerals, antioxidants, and probiotics have been encapsulated within hydrogels, which improve the stability of these compounds and control release routes in the digestive system [37]. In addition, hydrogels can protect bioactive compounds from denaturation or degradation during processing (such as high temperature and exposure to light). Bioactive compounds sensitive to various factors (e.g., anthocyanins, β-carotene, quercetin, and curcumin) that can cause their degradation should be protected by incorporating them into a hydrogel carrier. This requires adjusting the induction method, which includes modulating pH and ionic strength, to design carriers tailored to specific bioactive substances [37,38]. Furthermore, the pH and ionic strength of the final food product can also critically affect the properties of the hydrogel structure, which can consequently affect the bioactive functionalities of the embedded substances. 

Combining pea protein and psyllium shows great synergistic potential in producing a binary hydrogel with structural, textural, and nutritional advantages. Pea protein can contribute to the hydrogel’s structural integrity due to its inherent gelling and thickening properties. Psyllium can enhance the water-binding properties of the matrix, contributing to the overall stability of the hydrogel [8,39]. Despite that, there is limited information on pea protein–psyllium-based binary hydrogels, especially in the context of their properties under different pH and ionic strength conditions. This research aims to evaluate the induction conditions, such as the influence of pH and ionic strength on the physical stability and mechanical properties of binary pea protein–psyllium hydrogel for delivering bioactive compounds.

## 2. Results and Discussion

**Figure 1:**
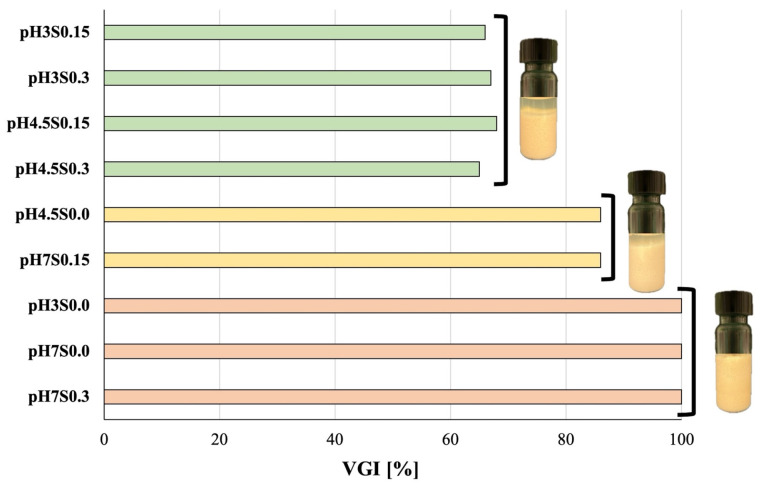
The volumetric gelling index (VGI) of the analyzed hydrogels and the appearance of the hydrogels in the vials. The columns differentiated by color differ significantly (*p* ≤ 0.05).

The volumetric gelling index (VGI) was calculated to determine the effect of pH and NaCl concentration modulation on the formation of a gel structure. Figure 1 depicts the VGI values and the appearance of the analyzed hydrogels. 

Based on VGI values, it can be observed that the sample induced at pH 7 without the addition of NaCl entirely formed a gel structure. At low pH and without the addition of NaCl, only the hydrogel obtained at pH 4.5 did not form a complete gel structure. However, the increase in ionic strength at pH 7 only affected the gel structure when the addition of NaCl was equal to 0.15 M, while the addition of 0.3 M NaCl did not affect the value of VGI (100%). Moreover, increasing the ionic strength at lower pH values decreased this value to ~65% significantly. This effect might be due to the higher rate of interactions between the proteins, which is caused by the change in the ionic strength of the polymeric chains. Consequently, these interactions affected the formed structure by expelling a certain amount of water in the syneresis process [5]. 

For the hydrogels obtained without the addition of NaCl, the decrease in the pH value from 7 to 4.5 affected L* by significantly increasing its value to 75.40 (Table 1). However, a further reduction in the pH to 3 decreased the value of L*. The same trend was observed in the case of the hydrogel obtained with the addition of NaCl (0.15 M). On the other hand, 0.3 M NaCl did not affect the value of L* at any pH. Based on the η2 coefficient, it can be concluded that both pH values and NaCl addition had a strong effect on L* variance. The values of a* increased significantly when the pH decreased from 7 to 3 for both hydrogels without and with the addition of NaCl (0.15 M). Furthermore, the b* values increased when decreasing the pH from 7 to 4.5 for hydrogels induced at high ionic forces (0.15 and 0.3 M NaCl), and then the b* values dropped when reaching pH 3 for all the hydrogels. In the case of the b* parameter, NaCl addition had the most potent effect on the variance (η2 = 0.958). These interdependencies were also observed in the case of the whiteness index (WI) and the yellowness index (YI), where the NaCl concentration had the highest effect on these color parameters at each tested pH. The decrease in the whiteness and increase in the yellowness of the analyzed hydrogels can be due to changes in the protein conformation and the interactions between protein molecules, which, in turn, affect their solubility. This was also observed by O’Flynn et al. [40] in their studies on the solubility of a commercial soy protein isolate at pH values of 2.0, 6.9, and 9.0. Thus, it can be stated that at a pH lower than the isoelectric point, the solubility of proteins can be decreased, which might affect the color parameters of the analyzed sample [41,42]. 

The color difference parameter (∆E) was calculated to comprehensively determine the effect of pH and NaCl concentration modulation on the obtained hydrogels (Table 2). In most samples, it was found that the difference between the obtained hydrogels higher than 5, meaning that the observer could notice two different colors [43]. Reducing the pH from 7 to 3 had a more significant effect on ∆E values in hydrogels obtained without the addition of NaCl (∆E in the range of 3–3.5) compared to those obtained with the addition of NaCl, 0.15 M (∆E ~ 2) and up to 0.3 M (∆E in the range 0.5–15). However, when analyzing the effect of increasing the ionic strength in hydrogels obtained at the same pH, it was observed that a more significant change in the values of ∆E occurred at low pH, 3 and 4.5 (∆E in the range of 7–8) compared to pH 7 (∆E ~ 5). 

The instability index was computed to assess the physical stability of the obtained hydrogels. This index ranges from 0 for stable to 1 for unstable samples. Table 3 shows the mean values of the instability index for each tested hydrogel. Both pH and salt concentration modulation significantly affect the physical stability of the analyzed hydrogels. Lowering the pH value from 7 to 4.5 (without modulating the ionic strength) increased the instability index value, indicating that the stability of the hydrogel decreased. However, when the pH reached 3, the instability index value significantly dropped. The increase in the ionic strength caused the value of the instability index to rise for the hydrogel induced at pH 7 while causing a drop in the index values for the hydrogel at pH 4.5. A similar trend was observed in the case of lowering the pH from 7 to 3 with the addition of 0.15 M NaCl. Nonetheless, a reversed effect was observed when the pH was decreased from 7 to 4.5 for hydrogels with the addition of 0.3 M NaCl, where both hydrogels at pH 4.5 and 3 had significantly higher stability. This effect could be due to forming a more robust gel structure caused by this ion modulation, which affected the interaction between the biopolymers by reducing the repulsion between them. Lei et al. [44] made a similar observation, in the case of walnut protein–κ–carrageenan composite gels, where the addition of Na^+^ significantly improved the bond strength between the biopolymers, resulting in a much denser and more uniform gel structure with improved water-holding capacity. In a study concerning the effect of adding NaCl on the thermal aggregation and gelation of soy protein isolate, Chen et al. [45] concluded that increasing the ionic strength, in many aspects, is similar to decreasing the pH value of the system. Moreover, the addition of NaCl affected the size and density of the elementary protein aggregates, leading to a more heterogeneous microstructure, which can decrease the water-holding capacity of such hydrogels. 

When the texture measurement data were analyzed (Table 3), it was found that only the transition from pH 4.5 to 3 increased the strength value (the force needed to immerse the texture analyzer probe into the hydrogel structure) of the analyzed hydrogels. The strength values significantly decreased when changing the pH from 7 to 4.5 for the hydrogels not containing NaCl. Moreover, by increasing the NaCl content, the strength of the hydrogels induced at pH 3 proportionally increased, while in the case of pH 7 and 4.5, the strength value only increased when 0.15 M NaCl was added. Further NaCl addition (0.3 M) caused a significant drop in strength for the hydrogels induced at pH 7. Both pH and NaCl addition strongly affected the strength variance (η2 = 0.930). The increase in strength could be due to the strengthening of electrostatic forces, hydrophobic interactions, hydrogen bonds, and disulfide bonds between the biopolymer chains, resulting in a more rigid microstructure. In the case of adhesion, which shows the force needed to separate the analyzer probe from the hydrogel, NaCl addition had a nonsignificant effect compared to pH on the variance of this parameter. A downward trend in the adhesion values was observed when decreasing the pH from 7 to 4.5 (hydrogels induced without NaCl) and from 7 to 3 (hydrogels with the addition of 0.15 M NaCl). However, the decrease in pH for the hydrogels induced with the addition of 0.3 M salt did not affect the adhesion values. The lowest adhesion value was recorded in the case of the hydrogel induced at a pH of 4.5 and without NaCl addition. This effect might be due to the low attractive and repulsive forces (no net charge) observed by Schuldt et al. [46] in their studies on NaCl and acid-induced soy protein hydrogels. 

In the case of spreadability, when decreasing the pH from 7 to 4.5 (for all the hydrogels with and without NaCl addition), an increase in the value of spreadability was noticed, meaning that these hydrogels needed higher job input to be spread evenly between two surfaces. Moreover, a further decrease in pH (from 4.5 to 3) in the case of hydrogels obtained with salt (0.15 M) caused a drop in the value of spreadability. On the other hand, a rise in the analyzed value was noticed in the case of hydrogels obtained with the addition of 0.3 M NaCl. The high spreadability value of the hydrogels obtained at pH 3 and 4.5 with the addition of NaCl could be attributed to the formation of protein aggregates and interactions between them. However, the hydrogels obtained at pH 7 displayed inferior spreadability and high adhesion values due to the higher unbound water [47,48].

**Figure 2:**
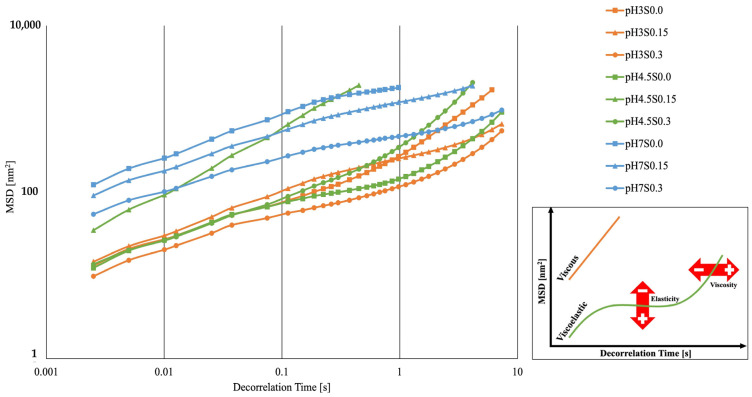
Mean Square Displacement (MSD) as a function of decorrelation time for the obtained hydrogels. The values presented in the figure are mean values (*n* = 3).

By analyzing the solid–liquid balance index (SLB) and elasticity index (EI) values (Table 3), it was noticed that both pH and ionic strength significantly affect the microrheological properties of the obtained hydrogels. NaCl had a more substantial effect on the variance of SLB (η2 = 0.838) while pH had a stronger effect on the variance of EI (η2 = 0.959). Nonetheless, both parameters affected the elasticity index of the obtained hydrogels. The decrease in the pH value from 7 to 4.5 shifted the properties of the hydrogel (induced without the addition of NaCl) to more solid-like (SLB < 0.5). However, a further decrease in the pH value to 3 caused the hydrogel to exhibit more viscous (liquid-like) properties (SLB > 0.5). Furthermore, the addition of salt changed this trend, causing the SLB index of hydrogels at pH 4.5 to reach higher values, with the hydrogel at pH 4.5 and the addition of 0.15 M NaCl reaching the highest value. NaCl (0.3 M) at pH 7 and 3 caused the hydrogels to exhibit highly solid-like properties. In the case of the EI, it was observed that only the hydrogel induced with the addition of salt exhibited higher elasticity when the pH value decreased [46,49]. Based on the analysis of the average trajectory of numerous particles in the obtained hydrogels, the Mean Square Displacement (MSD) was obtained as a function of decorrelation time (Figure 2). The analyzed samples had mostly viscoelastic properties, exhibiting the properties of soft gels (weak gels). The non-rectilinear MSD curve moving close to the baseline indicates that more particles were immobilized (caged) by the obtained gel network. The hydrogel induced at pH 3 and 0.3 M NaCl exhibited predominantly viscoelastic properties. On the contrary, the hydrogel induced at pH 7 and 4 (with 0.15 M NaCl) presented the least viscoelastic properties—their MSD curves leaned towards becoming more rectilinear, presenting more viscous characteristics.

**Figure 3:**
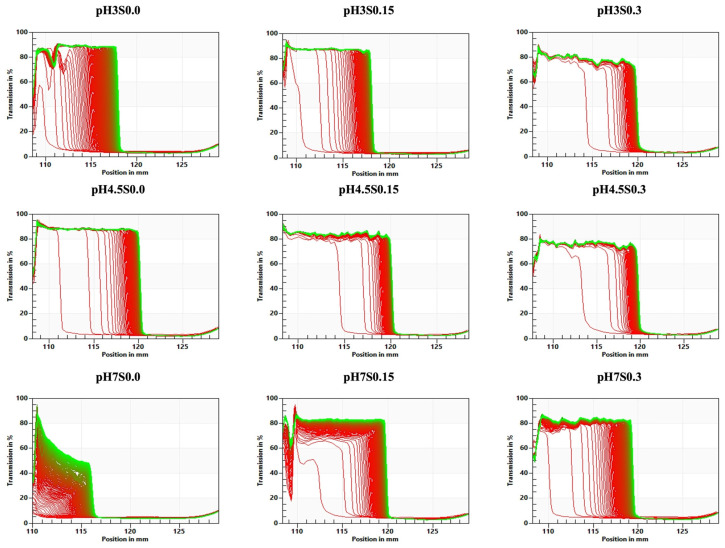
Evolution of transmission profile “fingerprints” of the obtained hydrogels. The red lines indicate light extinction at the starting point, and the green lines represent light extinction at the end of the analysis.

The transmission profiles obtained through STEP technology (space-time resolved extinction profiles) serve as ‘fingerprints’, indicating variations in particle concentration within the analyzed samples (Figure 3). For each obtained hydrogel, the changes in transmission profiles offer crucial insights into the kinetics of particle concentration fluctuations caused by the centrifugal forces leading to phase separation. After subjecting the hydrogel samples to centrifugal force, structural compression was observed. This compression is depicted by a decrease in light transmission in the right corner of each graph. The destabilization front moved downward in the sample, meaning that the highest particle concentration was at the bottom of the sample. At the same time, a transparent water phase remained in the upper parts [50,51]. Based on the transmission profiles, it is possible to conclude that the slowest destabilization changes occurred in the hydrogel induced at pH 7 (without the addition of salt). The structural compression was more significant when the pH was decreased from 7 to 4.5 (for all the hydrogels). However, the quickest compression occurred in the hydrogel induced at pH 4.5 without NaCl. Moreover, the hydrogels obtained with the addition of 0.3 M NaCl (at each of the pH values) were less affected by the centrifugal force applied during the analysis than the other hydrogels.

**Figure 4:**
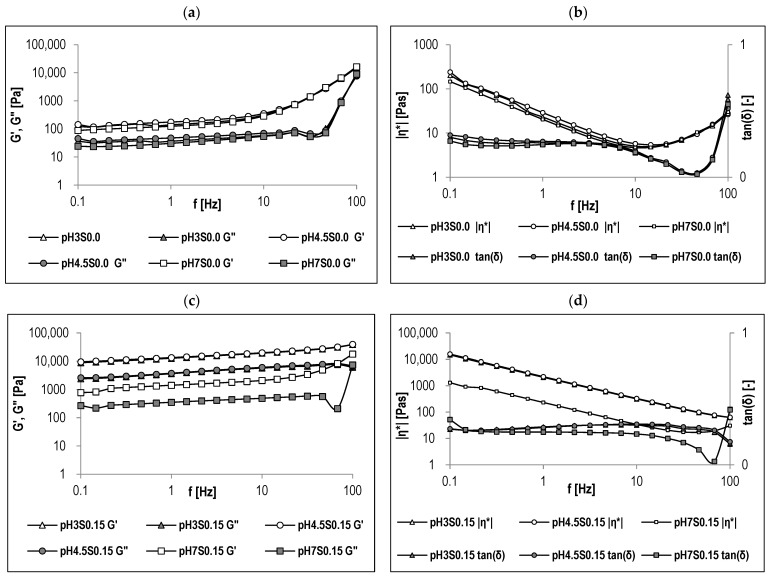

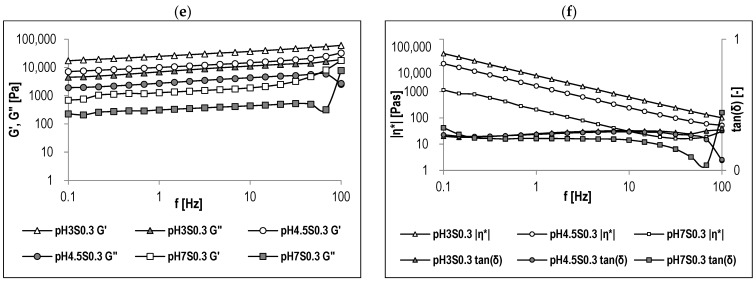
Mechanical spectra of the obtained hydrogels are presented graphically as functional relationships G′, G″, |η*|, and tan(δ) = f(Hz). (**a**,**b**) without NaCl, (**c**,**d**) with 0.15 M NaCl, (**e**,**f**) with 0.3 M NaCl (*n* = 3). G′—storage (elastic) modulus; G″=—viscous (loss) modulus; |η*|—complex viscosity; tan(δ)—degree of viscoelasticity.

Because hydrogels are viscoelastic systems, analyzing their rheological character under dynamic shear conditions provides information on their nature and behavior under slight deformations. The parameters determining a system’s rheological character are its primary structure, external factors, and observation time. As a result, dynamic (oscillatory) rheology tests are used to understand this characteristic, which is correlated with the degree of cross-linking and heterogeneity of the biopolymeric network [52,53]. Figure 4 showcases the evolution of mechanical spectra G’, G”, |η*|, and tan(δ) as a function of frequency (f). These values are displayed on a logarithmic-logarithmic graph, showcasing three decades of oscillation frequency, ranging from f = 0.1 to 100 Hz. These mechanical profiles were acquired within the linear viscoelastic range (1% deformation), preserving the original structure of the samples. Frequency sweep is a valuable tool for evaluating a sample’s viscoelastic attributes across varying timescales. Moreover, it facilitates the determination of critical parameters such as the storage (elastic) modulus (G’) and the viscous (loss) modulus (G”). The storage modulus (G’) gauges a material’s elastic behavior by quantifying the energy stored during shear. In contrast, the viscous modulus (G”) assesses its viscous response by measuring heat dissipation. The resulting storage and loss moduli offer valuable insights into how a material will perform in various applications, helping industries design products with specific mechanical properties and desired performance under different stress conditions [54,55]. Based on the presented spectra (Figure 4a,c,d), it can be observed that all the obtained hydrogels present viscoelastic characteristics specific for a weak gel system (such as ketchup, yogurt, custard) [56] since G’ remains above G” over the experimental frequency range, the separation of the G’ and G” curves is less than one decade, and tan(δ) reaches values higher than 0.2. Moreover, it can be concluded that hydrogels formed with the addition of NaCl, compared to those without NaCl, show greater gel strength or cross-linking density regardless of pH because both moduli assume higher values and are almost independent of frequency (Figure 4c,e). In weaker gels formed without NaCl, the mechanical spectra show some dependence on the oscillation frequency, and the range of the elastic plateau occurs at lower oscillation frequencies, up to frequencies where tan(δ) begins increasing (Figure 4a,b). Furthermore, the complex viscosity |η*| (Figure 4b,d,f) decreased with increasing frequency in the frequency range of the elastic plateau, indicating that the investigated hydrogels exhibit shear-thinning behavior. The available studies have also reported similar rheological characteristics of such binary protein–polysaccharide hydrogels [57,58]. Moreover, Zhang et al. [59], in their studies on the rheology and microstructural properties of gelatin-tara gum hydrogels, stated that salt addition affects the gel network structure by screening interactions, leading to a notable decrease in rheological properties. The most pronounced effect occurs at NaCl concentration ~50 mmol/L, due to the salting-in effect. However, they observed that higher salt concentrations significantly increase gel strength and prevent phase separation at low pH.

The hydrogels’ LVR (linear viscoelastic region) occurs at low shear stress when the moduli are independent of increasing stress (the system’s response is independent of the deformation magnitude, and the structure is maintained intact). Because structural properties correlate well with elasticity, the length and value of the LVR of the elastic modulus (G’) can be used to assess sample structure stability [60,61]. The values of LVR determined in this study are presented in Table 4. Decreasing the pH affected the G’ value significantly only in the case of hydrogels induced with NaCl (η2 = 0.940). This tendency was not observed in the case of the samples induced at a pH value of 7. Additionally, the length of LVR represented by γ (%) indicates the structural stability of the analyzed systems. It was observed that when decreasing the pH from 7 to 4.5, the length of LVR was reduced, and after reaching pH 3, this length increased again. This trend was observed in all the obtained hydrogels, regardless of the NaCl addition. However, salt addition significantly increased the γ value, which might be due to the formation of a more robust gel network. Nonetheless, in the case of the samples induced at pH 4.5, only the addition of the highest NaCl concentration (0.3 M) affected the system’s stability, which is also in correlation with what was observed in the case of the instability index (Table 4, Figure 1 and Figure 2). 

Based on the values of G’, G”, |η*|, and tan(δ) at 1 Hz (Table 4), it can be concluded that both pH and NaCl concentration have a significant effect on the variance of the parameters. The effect of decreasing the pH for the samples induced without salt on the values of G’ and G” was nonsignificant. However, when the samples were induced with 0.15 M NaCl, both moduli values were increased when the pH dropped from 7 to 4.5. In the case of hydrogel induction with the addition of 0.3 M NaCl, the change from pH 7 to 3 significantly increased the values of G’ and G” (reaching the highest values at pH 3). Additionally, at pH 3, the increase in the NaCl addition from 0 to 0.15 M caused the moduli to increase by 95 times on average, while the increase from 0 to 0.3 M caused the moduli to increase by 170 times. Zhu et al. [16] studied strong and elastic pea protein hydrogels formed using the pH-shifting method. They found that this method altered the protein chain structure, creating more active sites that enhanced intermolecular interactions. Moreover, studies on the impact of citric acid concentration and pH on the mechanism and rheological properties of whey protein hydrogels concluded that the induction of protein hydrogel at a low pH value affected the formation of the gel network, resulting in a more rigid hydrogel [62]. Furthermore, Tanger et al. [5], in their study on the effect of pH and ionic strength on the thermal gelation behavior of pea protein, noticed that the addition of salt at pH 3 led to the development of a stiffer gel structure caused by a high entanglement of the protein. The degree of viscoelasticity, tan(δ), is defined as the ratio of G”/G’. When the analyzed systems exhibit solid-like (elastic) properties, tan(δ) is lower than 1. However, for systems with more liquid-like (viscous) properties, tan(δ) is higher than 1 [63]. In the case of the obtained hydrogels, all the variants had a tan(δ) value lower than 1 at at 1 Hz, ranging from 0.24 to 0.29, meaning that the formed hydrogels left at rest have properties similar to a solid but can be easily spread, such as yogurts, ketchup, and jams.

**Figure 5:**
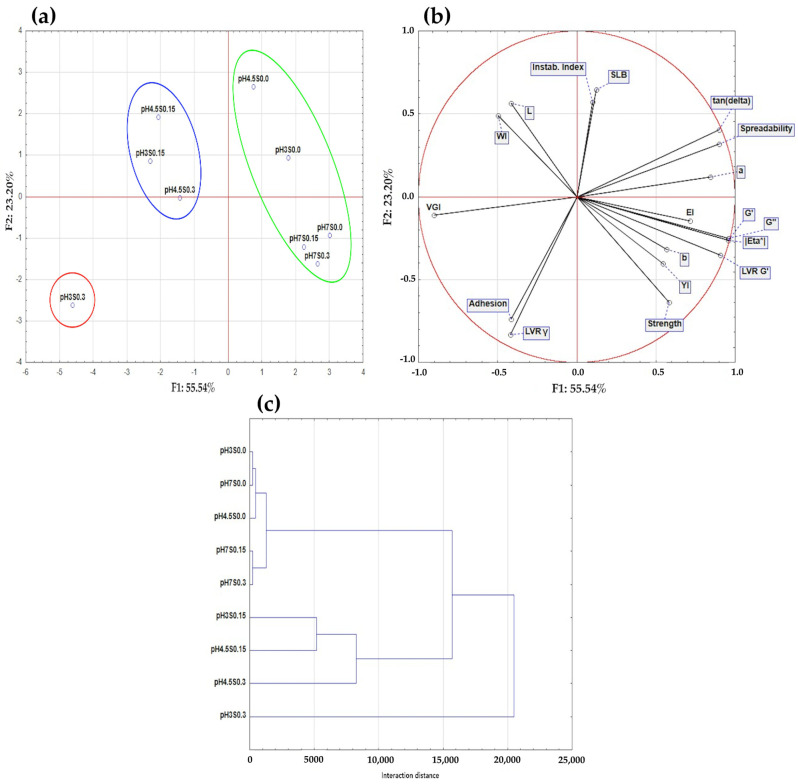
Principal component analysis PCA: (**a**) score plot, F1 versus F2 of all samples. (**b**) Score plot, F1 versus F2 of data from determinations used as variables. and hierarchical cluster analysis HCA (**c**) of the obtained hydrogels.

The obtained results were subjected to a principal component analysis (PCA) and a hierarchical cluster analysis (HCA). These two statistical methods were chosen to effectively summarize the data gathered in this study. The PCA was conducted with 12 active variables. Two principal components were identified (Figure 5a,b): Component 1 (F1) explained 55.54% of the variance, and Component 2 (F2) explained 23.20% of the variance. Combined, these two components account for a total of 78.74% of the variance in the results. Component 1 is strongly positively correlated with VGI (r = 0.90). On the other hand, a negative contribution of this factor was found for G’ (r = −0.95), G” (r = −0.96), |η*| (r = −0.95), and spreadability (r = −0.90). Component 2 is positively correlated with the instability index (r = 0.64) and negatively correlated with γ (r = –0.80) and adhesion (r = −0.70). Considering the above interdependencies, the first principal component can be interpreted as a measure of the conditions under which the hydrogel can form a gel structure with high elastic properties. In contrast, the second component can be interpreted as a measure of the conditions under which the hydrogel can form a structurally stable system. Based on the hydrogels’ distribution across the space of the principal components (Figure 5a) and the interaction distances shown in HCA (Figure 5c), it can be concluded that the first and the most prominent cluster on the right from the center (green cluster) represents the hydrogels with the highest volumetric gelling index values but low G’ and G” values (less elastic). These hydrogels were induced at pH 7, 4.5, and 3 (without NaCl addition) and pH 7 (with 0.15 and 0.3 M NaCl). Additionally, at pH 4.5 and 3 (the upper part of the green cluster), it was possible to obtain hydrogels with lower structural stability. The next cluster is the one in the upper left from the center (blue cluster), showcasing that the induction at pH 4.5 (0.15 and 0.3 M NaCl) and pH 3 (with 0.15 M NaCl) results in hydrogels with high G’ and G” moduli but low structural stability and VGI. However, only the sample induced at pH 3 and 0.3 M NaCl (pH3S0.3) showed the most significant difference from the other hydrogels (highest interaction distance, Figure 5c). The hydrogel exhibited the most elastic properties at pH 3 and 0.3 M NaCl and had high structural stability. Nonetheless, it still exhibited a low VGI value, which might have been caused by the high interactions between the biopolymers leading to the loss of a certain amount of water. 

## 3. Conclusions

To summarize, altering pH and ionic strength resulted in pea protein–psyllium hydrogels with varied properties, all characterized as weak gels. Lower pH levels (notably pH 3 and 4.5) led to significant color changes and produced weak, easily spreadable gels without NaCl. Adding NaCl (0.15 and 0.3 M) at these pH levels improved the hydrogels’ structural stability and moduli, with the hydrogel at pH 3 with 0.3 M NaCl showing the highest elasticity and stability. This study demonstrates a critical relationship between pH and ionic strength, where NaCl’s impact during hydrogel induction highly depends on the pH. The industrial utility of these hydrogels is determined by their inherent properties, which dictate their specific applications. Hydrogels produced under low pH and high ionic strength conditions are promising delivery systems for low-pH-stable bioactive compounds, including anthocyanins. Further research is essential to validate their potential as food texture enhancers and effective delivery systems.

## 4. Materials and Methods

### 4.1. Materials

Pea protein (NUTRALYS^®^ F85F, protein content 88%, ash 10%) was obtained from Roquette Freres (Lestrem, France). Psyllium husk powder (PS, type 10351, purity 95%, particle size 60 mesh–250 µm) was obtained from C.E. Roeper GmbH (Hamburg, Germany). Citric acid (purity ≥ 99.5%) and sodium citrate (purity 95%) were purchased from the local food ingredient supplier Agnex (Białystok, Poland).

### 4.2. Hydrogel Induction 

The investigated hydrogels’ induction involved pea protein hydration (12.5 g of protein/100 g) in distilled water while stirring for 60 min, using a heating magnetic stirrer (300 RPM). The obtained protein dispersion was heated to 80 °C for 30 min. After cooling the dispersion to 20 °C, psyllium husk was added (concentration of 0.5 g/100 g), and the dispersion was stirred for 10 min (300 RPM). The pH of the dispersions (from 7 to 4.5 and 3) was adjusted using 1 M citric acid and 1 M sodium citrate solutions. The ionic strength of the dispersion was modified by adding NaCl (to 0.15, 0.3 M) (Table 5). Then, the obtained dispersions were stored for 24 h at 4 ± 1 °C to develop a gel structure.

### 4.3. Methods

#### 4.3.1. Volumetric Gelling Index (VGI)

The VGI was used to determine the degree of hydrogel formation. It is a parameter that expresses a dispersion’s ability to form a gel structure. The VGI equals zero when no gel structure is formed and 100% when the sample is completely gelled. The VGI is calculated using the equation below [50].

(1) $$VGI=\frac{V_{G}}{V_{T}}\cdot 100,$$

where V_G_—volume of the formulated gel and V_T_—total volume of the sample. The reported values represent the averages of three replicates.

#### 4.3.2. Color Parameters 

A Minolta CR-5 colorimeter (Minolta, Japan; light source D65; measuring head hole: 8 mm) was used to measure the color components in the CIE L* a* b* system at the surface of the obtained hydrogels. The total color difference (∆E), whiteness (WI), and yellowness index (YI) indexes were calculated using the obtained L*, a*, and b* parameters. ∆E was computed to determine the color differences between the obtained hydrogels. The total color difference ∆E was calculated based on the following equation [43]:

(2) $$∆E=\sqrt{{\left(L_{s1}^{*}-L_{s2}^{*}\right)}^{2}+{\left(a_{s1}^{*}-a_{s2}^{*}\right)}^{2}+{\left(b_{s1}^{*}-b_{s2}^{*}\right)}^{2}},$$

where L*_S1_; a*_S1_; b*_S1_ and L*_S2_; a*_S2_; b*_S2_ refer to the color parameters of the compared hydrogels. The whiteness (WI) and yellowness (YI) index were calculated using the following equations [64]:

(3) $$WI=100-\sqrt{{\left(100-L*\right)}^{2}+{a*}^{2}+{b*}^{2}},$$

(4) $$YI=142.86\cdot \frac{b*}{L*},$$

where L*, a*, and b* refer to the color parameters of each analyzed hydrogel. The reported values represent the averages of three replicates.

#### 4.3.3. Physical Stability and Destabilization Behavior

LUMiSizer 6120-75 (L.U.M. GmbH, Berlin, Germany) was used to assess the physical stability and destabilization kinetics of the obtained hydrogels. This assessment method involved centrifuging the hydrogels while illuminating the entire sample cell with near-infrared (NIR) light—STEP technology (Space and Time Extinction Profiles). The sensor measures the intensity of transmitted light as a function of time and position over the entire sample length, and the data are converted and recorded using the provided software (SepView 6.0; LUM, Berlin, Germany) [65]. Before running this analysis, the following parameters were established: dispersion volume 1.8 mL, wavelength 870 nm, light factor 1, 1500 rpm, experiment period 15 h 10 min, interval time 210 s, and temperature 20 °C. The destabilization behavior (fingerprint) was obtained from the recorded data, and the instability index was calculated. The values reported are the averages of three replicates.

#### 4.3.4. Textural Properties

The texture analysis was performed using a texture analyzer (TA.XT Plus, Stable Micro Mixtures, Surrey, UK) with a 5 kg load cell. The texture analyzer was equipped with a 0.5 cm diameter cylindrical flat probe (P/0.5R) to measure the strength (N) and adhesion (N) of the hydrogels. The sample penetration depth was set at 8 mm, the measurement speed was 1.0 mm/s, and the temperature was 20 °C. To measure the spreadability (N·s) of the obtained hydrogels, the texture analyzer was equipped with a TTC Spreadability Rig. The measurement speed was set at 3.0 mm/s. The gathered data were processed with the Exponent version 6.1.4.0 (Stable Micro Mixtures, Surrey, UK) equipment software. The values reported are the averages of three replicates. 

#### 4.3.5. Microrheological Properties

The microrheological properties of the hydrogels were investigated using a Rheolaser Master device (Formulaction, L’Union, France). The device operates in the near-infrared region (wavelength of 650 nm) using the dynamic MS-DWS (Multi Speckle Diffusing Wave Spectroscopy) technique. The interfering backscattered waves are captured by the detector, and the measurement results are recorded using Rheotest software 1.4.0.11. The following microrheological parameters were determined using the raw data obtained: mean square displacement (MSD) curves, elasticity index (EI) (nm^−2^), and solid-liquid balance (SLB). MSD represents the mean of several scattering trajectories of particle movement as a function of the time of the analyzed hydrogel. SLB is the ratio (G’/G”) of the elastic modulus to the viscous modulus. The reciprocal of the MSD value at the plateau is used to calculate EI, which is directly proportional to the elastic modulus (G’) [50,66]. The values that are reported are the averages of three replicates.

#### 4.3.6. Rheological Properties

A Haake Mars 40 rheometer (Thermo Scientific, Karlsruhe, Germany) was used to measure the rheological properties of the obtained hydrogels. A plate (35 mm in diameter, 1 mm gap) with serrated platens was used, and their temperature was kept at 20 °C. Two different small-amplitude oscillatory shear tests were performed (20 °C): strain sweep and frequency sweep. The strain amplitude test was performed at a constant frequency of 1 Hz with a strain ranging from 0.1 to 100% to identify each hydrogel’s linear viscoelastic region (LVR). The frequency sweep test was then performed with a frequency range of 0.1–10 Hz and a constant strain of 1%. The frequency sweep test was used to determine the parameters describing the viscoelastic behavior of the samples, which included the elastic modulus (G’), viscous modulus (G”), complex viscosity (|η*|), and loss angle tan(δ) as the ratio of G” to G’. The values reported are the averages of three replicates.

#### 4.3.7. Statistical Analysis

The data acquired from the experiments were analyzed using Statistica 13.1 (StatSoft, Krakow, Poland). The effects of pH and NaCl concentration modulation on the experiment’s observed results were determined using analysis of variance (ANOVA). Tukey’s test was used to determine the significance of the differences at α = 0.05. The results were also evaluated using principal component analysis (PCA) and hierarchical cluster analysis (HCA).

## Figures and Tables

**Table 1:** Color parameters of the analyzed hydrogels (*n* = 3).

Samples	Color Parameters				
	L*	a*	b*	WI	YI
pH3S0.0	73.85 ^e^ ± 0.07	4.50 ^cd^ ± 0.07	17.12 ^a^ ± 0.05	68.42 ^d^ ± 0.04	33.12 ^a^ ± 0.06
pH3S0.15	67.70 ^a^ ± 0.11	5.60 ^e^ ± 0.03	21.40 ^c^ ± 0.02	60.86 ^ab^ ± 0.08	45.15 ^c^ ± 0.04
pH3S0.3	68.07 ^b^ ± 0.12	5.28 ^de^ ± 0.05	22.28 ^cd^ ± 0.24	60.71 ^ab^ ± 0.23	46.76 ^cd^ ± 0.57
pH4.5S0.0	75.40 ^f^ ± 0.06	3.41 ^ab^ ± 0.07	19.37 ^b^ ± 0.15	68.51 ^d^ ± 0.06	36.70 ^b^ ± 0.25
pH4.5S0.15	69.11 ^c^ ± 0.13	5.35 ^de^ ± 0.04	22.41 ^cd^ ± 0.05	61.47 ^b^ ± 0.13	46.32 ^cd^ ± 0.18
pH4.5S0.3	68.12 ^b^ ± 0.03	5.16 ^de^ ± 0.01	22.80 ^d^ ± 0.02	60.47 ^a^ ± 0.01	47.82 ^d^ ± 0.02
pH7S0.0	72.02 ^d^ ± 0.02	2.49 ^a^ ± 0.03	19.19 ^b^ ± 0.04	65.98 ^c^ ± 0.03	38,06 ^b^ ± 0.08
pH7S0.15	68.03 ^b^ ± 0.08	3.44 ^ab^ ± 0.04	21.50 ^c^ ± 0.05	61.32 ^ab^ ± 0.09	45.16 ^c^ ± 0.15
pH7S0.3	68.04 ^b^ ± 0.24	3.86 ^bc^ ± 1.06	21.66 ^cd^ ± 1.22	61.18 ^ab^ ± 0.88	45.49 ^cd^ ± 2.61
Statistic ANOVA, η2 [-]					
pH	0.979	0.881	0.696	0.548	0.542
NaCl	0.999	0.820	0.958	0.994	0.977
pH·NaCl	0.977	ns	0.653	0.871	0.692

a, b, c…—mean values in columns differentiated by letters differ significantly (*p* ≤ 0.05). η2—coefficient indicating the extent of the effect of factors, pH, NaCl concentration, and pH·NaCl. ns—nonsignificant. WI—whiteness index; YI—yellowness index.

**Table 2:** The color difference parameter (∆E) between the analyzed hydrogels.

Samples	pH3S0.0	pH3S0.15	pH3S0.3	pH4.5S0.0	pH4.5S0.15	pH4.5S0.3	pH7S0.0	pH7S0.15	pH7S0.3
pH7S0.3	7.40	1.79	1.55	7.72	1.98	1.73	4.88	0.45	-
pH7S0.15	7.37	2.18	1.99	7.68	2.37	2.15	4.71	-	
pH7S0.0	3.42	5.76	5.74	3.51	5.20	5.95	-		
pH4.5S0.3	8.10	1.53	0.54	8.24	1.09	-			
pH4.5S0.15	7.15	1.75	1.05	7.25	-				
pH4.5S0.0	2.94	8.26	8.11	-					
pH3S0.3	7.79	1.01	-						
pH3S0.15	7.57	-							
pH3S0.0	-								

The values presented in that table are mean (*n* = 3). Depending on the ΔE values the color difference between the analyzed hydrogels can be estimated as not noticeable for the observer (0 < ΔE < 1), only an experienced observer can notice the color difference between the hydrogels (1 < ΔE < 2), an inexperienced observer can also notice the color difference (2 < ΔE < 3.5), clear color difference noticed (3.5 < ΔE < 5), and the observer can notice different colors (5 < ΔE).

**Table 3:** Instability index, textural properties, and microrheological properties of the analyzed hydrogels (*n* = 3).

Samples	Instability Index	Textural Properties			Microrheological Properties	
		Strength (N)	Adhesion * (N)	Spreadability [N·s]	SLB	EI × 10^−3^ (nm^−2^)
pH3S0.0	0.45 ^d^ ± 0.02	0.11 ^b^ ± 0.01	0.04 ^d^ ± 0.01	13.3 ^c^ ± 1.3	0.54 ^b^ ± 0.04	6.1 ^b^ ± 0.1
pH3S0.15	0.49 ^e^ ± 0.01	0.15 ^c^ ± 0.00	0.02 ^b^ ± 0.00	26.2 ^ef^ ± 0.8	0.53 ^b^ ± 0.04	7.9 ^bc^ ± 0.6
pH3S0.3	0.35 ^b^ ± 0.02	0.24 ^e^ ± 0.02	0.04 ^d^ ± 0.00	24.3 ^e^ ± 1.8	0.35 ^a^ ± 0.01	17.0 ^d^ ± 1.3
pH4.5S0.0	0.55 ^f^ ± 0.01	0.07 ^a^ ± 0.00	0.01 ^a^ ± 0.00	13.3 ^c^ ± 0.7	0.45 ^ab^ ± 0.04	9.8 ^c^ ± 1.7
pH4.5S0.15	0.45 ^d^ ± 0.01	0.15 ^c^ ± 0.00	0.03 ^bc^ ± 0.00	28.6 ^f^ ± 2.1	0.73 ^c^ ± 0.04	2.4 ^a^ ± 0.5
pH4.5S0.3	0.33 ^b^ ± 0.00	0.14 ^c^ ± 0.00	0.04 ^d^ ± 0.00	19.1 ^d^ ± 1.8	0.54 ^b^ ± 0.01	7.3 ^b^ ± 0.9
pH7S0.0	0.20 ^a^ ± 0.00	0.08 ^a^ ± 0.00	0.05 ^e^ ± 0.00	8.0 ± 0.6	0.50 ^b^ ± 0.04	1.3 ^a^ ± 0.1
pH7S0.15	0.48 ^e^ ± 0.01	0.18 ^d^ ± 0.00	0.06 ^e^ ± 0.00	3.5 ^a^ ± 0.2	0.49 ^b^ ± 0.06	1.5 ^a^ ± 0.2
pH7S0.3	0.40 ^c^ ± 0.01	0.15 ^c^ ± 0.01	0.04 ^d^ ± 0.00	5.7 ^ab^ ± 0.2	0.36 ^a^ ± 0.03	3.6 ^a^ ± 0.7
Statistic ANOVA, η2 [-]						
pH	0.952	0.912	0.942	0.980	0.771	0.959
NaCl	0.970	0.974	ns	0.909	0.838	0.912
pH·NaCl	0.989	0.930	0.906	0.933	0.804	0.927

a, b, c…—mean values in columns differentiated by letters differ significantly (*p* ≤ 0.05). * Absolute value |Adhesion|. η2—coefficient indicating the extent of the effect of factors, pH, NaCl concentration and pH·NaCl. ns—nonsignificant. SLB—solid–liquid balance index; EI—elasticity index.

**Table 4:** Rheological parameters of the obtained hydrogels under oscillatory testing at 20 °C, which includes LVR (linear viscoelastic region) values obtained in the amplitude sweep test at 1 Hz; elastic (G’) and viscous (G”) moduli, tan(δ), and complex viscosity η* values at 1 Hz obtained in the frequency sweep test at a strain of 1% (*n* = 3).

Samples	LVR		Frequency Sweep. Values at 1 Hz			
	G’ Plateau [kPa]	γ [%]	G’ (kPa)	G” (kPa)	tan(δ) (-)	|η*| (Pa·s)
pH3S0.0	0.37 ^a^ ± 0.05	2.18 ^ab^ ± 0.17	0.14 ^a^ ± 0.03	0.04 ^a^ ± 0.01	0.26 ^bf^ ± 0.01	23 ^a^ ± 4
pH3S0.15	15.7 ^b^ ± 1.6	2.70 ^bc^ ± 0.26	12.7 ^c^ ± 1.0	3.65 ^c^ ± 0.26	0.29 ^f^ ± 0.00	2100 ^c^ ± 160
pH3S0.3	36.8 ^d^ ± 6.8	3.13 ^cd^ ± 0.37	24.9 ^d^ ± 1.0	6.97 ^d^ ± 0.29	0.28 ^cde^ ± 0.00	4110 ^d^ ± 160
pH4.5S0.0	0.80 ^a^ ± 0.02	1.39 ^a^ ± 0.04	0.17 ^a^ ± 0.03	0.05 ^a^ ± 0.01	0.27 ^cf^ ± 0.00	29 ^a^ ± 0
pH4.5S0.15	10.7 ^b^ ± 2.6	1.68 ^a^ ± 0.33	13.4 ^c^ ± 1.7	3.79 ^c^ ± 0.50	0.28 ^de^ ± 0.00	2210 ^c^ ± 280
pH4.5S0.3	23.5 ^c^ ± 1.6	2.56 ^bc^ ± 0.05	10.1 ^b^ ± 0.9	2.76 ^b^ ± 0.23	0.27 ^cd^ ± 0.00	1660 ^b^ ± 140
pH7S0.0	0.14 ^a^ ± 0.04	3.71 ^de^ ± 0.59	0.12 ^a^ ± 0.05	0.03 ^a^ ± 0.01	0.25 ^ab^ ± 0.00	19 ^a^ ± 1
pH7S0.15	1.2 ^a^ ± 0.2	4.06 ^ef^ ± 0.34	1.42 ^a^ ± 0.18	0.35 ^a^ ± 0.04	0.25 ^ab^ ± 0.00	233 ^a^ ± 29
pH7S0.3	1.4 ^a^ ± 0.3	4.82 ^f^ ± 0.04	1.28 ^a^ ± 0.32	0.31 ^a^ ± 0.09	0.24 ^a^ ± 0.01	210 ^a^ ± 54
Statistic ANOVA, η2 [-]						
pH	0.917	0.939	0.982	0.982	0.936	0.982
NaCl	0.940	0.768	0.984	0.983	0.712	0.984
pH·NaCl	0.891	ns	0.978	0.978	0.718	0.978

a, b, c…—mean values in columns differentiated by letters differ significantly (*p* ≤ 0.05). η2—coefficient indicating the extent of the effect of factors, pH, NaCl concentration and pH·NaCl. ns—nonsignificant. G′ Plateau—elastic modulus at the plateau; γ—length of LVR; G′—storage (elastic) modulus; G″—viscous (loss) modulus; tan(δ)—degree of viscoelasticity; |η*|—complex viscosity.

**Table 5:** Explanation of hydrogel sample coding.

Samples Code	pH	NaCl Addition (M)
pH3S0.0	3	0.0
pH3S0.15	3	0.15
pH3S0.3	3	0.3
pH4.5S0.0	4.5	0.0
pH4.5S0.15	4.5	0.15
pH4.5S0.3	4.5	0.3
pH7S0.0	7	0.0
pH7S0.15	7	0.15
pH7S0.3	7	0.3

## Data Availability

The data used in this contribution are available upon request.
